# Cyclic Fatigue Resistance and Fractographic Analysis of Race and Protaper Rotary NiTi Instruments

**Published:** 2011-05-15

**Authors:** Shahram Azimi, Parisa Delvari, Hamid Cyrus Hajarian, Mohammad Ali Saghiri, Kasra Karamifar, Mehrdad Lotfi

**Affiliations:** 1. Department of Endodontics, Dental School, Islamic Azad University of Medical sciences, Tehran, Iran.; 2. Dentist, Private Practice, Tehran, Iran.; 3. Department of Dental Material, Dental School, Islamic Azad University of Medical sciences, Tehran, Iran.

**Keywords:** Fractographic Analysis, Cyclic Fatigue, Torsional Failure, Rotary NiTi Instrument

## Abstract

**INTRODUCTION:**

This study investigated the fatigue and fracture modes of RaCe and ProTaper rotary instruments.

**MATERIALS AND METHODS:**

Fatigue resistance was evaluated by rotating the files 30° or 60° and with 2 or 5mm radius of curvature. RaCe taper 06 size 25 and ProTaper F1 files (n=40) were used. The number of rotations to failure was analyzed by two-way ANOVA and independent sample t-test. Fracture surfaces were examined under a scanning electron microscope.

**RESULTS:**

Both files exhibited significantly more resistance to fracture when angle severity was reduced and increase in curvature radius (P=0.000). ProTaper demonstrated higher number of cycle of failure (P=0.0029) in one group (r=5mm, 60º). SEM observation revealed fatigue mark/features in 17 specimens, shear characteristics in 37 samples, and tensile overload in 26 samples.

**CONCLUSION:**

Radius of curvature was the main factor in torsional and fatigue failures.

## INTRODUCTION

The use of nickel-titanium (NiTi) superelastic wires for manufacturing rotary instruments has ushered a new era in endodontic practice. The NiTi intermetallic alloy is characterized by two key properties including shape memory and superelasticity. These two properties impart the ability to rotate through the bending demands of curvatures [1][2][3]. However, such instruments have a risk of separation in the clinical setting due to cyclic fatigue and/or torsional stresses [4][5][6].

Crack initiation and transgranular crack growth leading to fatigue failure occur through slipping bands mechanism and by repeated cyclic loading [6][7]. Torsional failure is the result of differences between the rotation rates of the engaged file tip and the coronal part of the rotating file[8][9].

Given that cyclic fatigue is the main cause of file separation [10][11][12], numerous studies have investigated the factors influencing the fatigue processes. It has been shown that instrument design, diameter and taper, configuration of the root canal, and operating speed and torque may all contribute to fatigue failures [13][14][15][16][17][18].

Work hardening of the alloy during manufacturing process leaves areas of brittleness which are prone to crack initiation [19][20], and may decrease the fatigue life of materials [21]. Electropolishing may enhance the fatigue resistance of files by removing surface irregularities and residual stresses [19][22].

To reduce the risk of torsional failure, varying the instrument’s taper over the length of cutting blades is recommended. This reduces the zone of file engagement in the root canal and decreases the risk of instrument fracture. This concept has resulted in the introduction of ProTaper brand (Dentsply Maillefer, Ballaigues, Switzerland). The manufacturer’s claim that it generates lower torque values [23].

Another way to prevent the file’s excessive engagement in the canal walls is to alternate the cutting blades. Alternating cutting edges in RaCe rotary file (FKG Dentaire, La-Chaux de Fonds, Switzerland) design constantly switches the helix angles of the blades as they rotate inside the canal [24].

Rotation of rotary files in curved canals can generate both fatigue and torsional failures. Various loads can produce different fracture surface textures. SEM observations of the fracture surface have revealed two main patterns: 1) flexural failure characterized by a surface perpendicular to the direction of the tensile stress, linear striations, and areas of crack initiation [25][26][27]; and 2) torsional fracture with appreciable plastic deformation and dimpling patterns [27].

The aim of this study was to compare the fatigue life of ProTaper and RaCe rotary NiTi instruments when rotating in two different angles and radii of curvature as well as their main failure modes.

## MATERIALS AND METHODS

An apparatus similar to a previous study was designed[28] which consisted of a frame that supported a handpiece and a stainless steel block with artificial canals matched for each instrument size and taper. The stainless steel block was lathed by a CNC device (Maho MH 400E, Deckel-Maho-Gildemeister, Bielefeld, Germany) to a 110mm×100mm×10mm dimension and 63-65 Rockwell hardened stainless steel block. Two different testing radii and angles were prepared. Test canals with a total length of 16mm, with either 30º or 60º curvature, and 2 or 5mm radius of curvature, were prepared. Phosphate buffer saline was used as a lubricant during instrumentation.

Two types of files including RaCe 25.06 and ProTaper F1 of 40 each were used in this study and they were rotated in simulated canals with two radii (2 and 5mm) and two angles of curvature (30º and 60º); this resulted in eight test groups (n=10).

**Figure 1:**
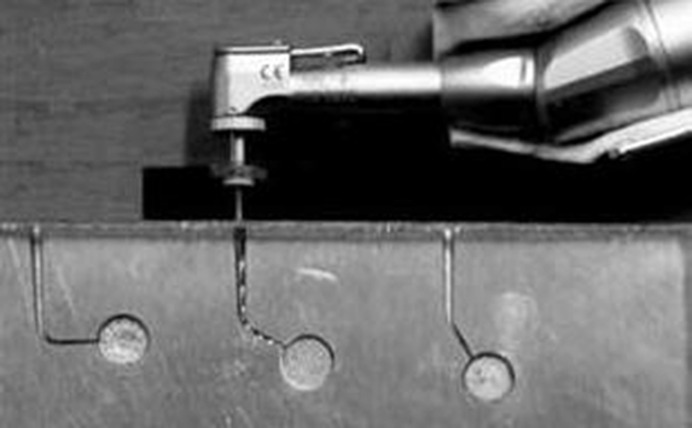
Fixed handpiece and rotating file in simulated metal block canal

Before receiving rotations, the instruments were introduced into the canals to the full working length by moving the blocks toward the fixed handpiece (Figure 1).

All the files were rotated at 300 rpm using a 1:16 reduction handpiece (Anthogyr, Sallanches, France) with a torque-controlled electric motor (TCM Endo; Nouvag, Goldach, Switzerland). The applied torque was 50 Nmm for RaCe and 70 Nmm for ProTaper, according to manufacturer’s instructions. The time to fracture was recorded by a chronometer accurate to 1:100 of a second and the number of rotations to breakage was calculated (NCF=time×speed).

Subsequently, fracture surfaces were examined under a scanning electron microscope (SEM) operating at 15 kV (Phillips XL30 FEG; FEI company, Hillsboro, VA, USA?).

Data were submitted to a 3-factor analysis of variance (ANOVA) to examine the effects of radius, angle, and file system. Two separate series of two-way ANOVA were carried out to examine the main effects of radius and angleon each file system. In addition, two more separate series of two-way ANOVA were performed to detect the main effect of angle and system at each radius. The interactions of the two file systems with each angle and the two curvature degrees with each file system were evaluated using Independent Sample t-test.

## RESULTS

Independent sample t-test showed that at 5mm radius and 60°, ProTaper (mean NCF of 184.5±29.8) outperformed RaCe (mean NCF of135±25.3) instruments, with significantly higher rotations to failure (P=0.0029). There was no significant difference between the two systems at 5mm radius and 30°, and between 2mm radius with either 60° or 30° angles.

A significant relationship between curvature degree and file system at 5mm radius (P=0.006) was found; two-way ANOVA test demonstrated significant failure differences for angle (P=0.000), and radius (P=0.000) with both RaCe and ProTaper groups.

SEM observation of fracture surfaces of the handle segments of files showed that from a total of 80 separated instruments, 21% (n=17) exhibited fatigue striations and crack initiation, indicating true fatigue failure (Table 1); and 79% (n=63) showed appreciable plastic deformation and dimpling patterns, suggesting torsional failure (37 samples had shear failure and 26 samples had tensile failure).

At the most severe curvature (2mm, 60°), all the specimens (n=20) followed shear process, whereas in the mildest curvature group (5mm, 30°), 15% (n=3) exhibited sheared specimens.

Within both RaCe and ProTaper files, two-way ANOVA test showed significant differences for angle (P=0.000) and radius (P=0.000).

## DISCUSSION

The primary objective of an instrument failure analysis is to determine the cause of failurewhich may be the result of design, manufacturing process, or complex canal configuration, and operator-related factors [29]. Fractures have been generally classified into ductile and brittle fractures. Ductile fractures are defined as slow tearing of the material with the expenditure of considerable energy, displaying a dimpled surface created by growth of internal voids of the metal and an appreciable gross plastic deformation. Brittle fracture is characterized by a rapid rate of crack propagation (due to fluctuating stress cycles), with no gross deformation [27]. Instruments with larger cross-sectional area and more voluminous inner core have more regular distribution of stresses and create more torque-resistant models [13][30]. However, our results showed that the ProTaper design with its convex triangular cross-section could undergo significantly greater rotation at 5mm radius and 60° angle compared to RaCe which has constant triangular cross-section.

**Figure 2:**
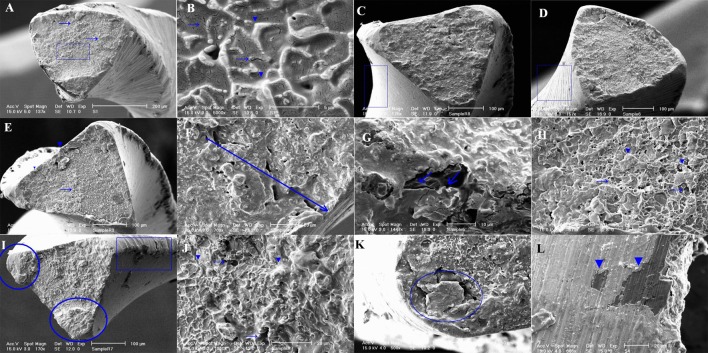
Separated endodontic files micrographs of a RaCe instrument arising from cyclic fatigue(A, B), 45⁰ titled (C, D), tensile overload (E, F, G, H), shear loads (I, J, K, L)

Figure 2 shows micrographs of a separated RaCe instrument arising from cyclic fatigue: A: the general view illustrating both fatigue (arrows) and dimpled (arrowhead) patterns (original magnification×153). B: presence of fatigue striations (arrowheads) and microcracks (arrows) can be noted on the same instrument indicating true fatigue failure (original magnification×7500).

In the 45° tilted samples: C: RaCe instrument with less surface irregularities. D: ProTaper file, showing rougher surface with deeply machined grooves and several pits on the blades.

Micrographs of separated instruments due to tensile overload: E: presence of necked regions (arrowheads) and microvoid coalescence (arrows) indicating tensile fracture. F: growth of microvoids from the center toward the surface on the same instrument. G: growth of void sheets adjacent to the surface, representing tensile failure. H: presence of both equiaxed dimples (arrowheads) and microvoid coalescence (arrows), indicating true tensile overload.

Micrographs of separated instruments arising from shear loads: I: plastic deformation (rectangle) and fracture at different planes (circles) due to final catastrophic failure on the same instrument. J: presence of skewed dimples (arrows) and tearing ridges (arrowheads) indicating true shear failure. K: failure of specimen into small pieces due to the angulation of shear plane to the axis of the specimen. L: longitudinal cracks indicating presence of shear loads.

SEM analysis revealed that all failures at 5mmradius and 60° curvature were torsional. No significant differences could be detected in fatigue resistance of the two instruments tested, while RaCe instruments showed insignificantly higher NCF rates at 30° angle (Table 1). These results are not compatible with pervious observations which found that files with larger cross-sections (e.g. ProTaper) succumb to fracture at higher NCF. However, these studies have concentrated only on cross-sectional geometry [13][33]. In addition, finite element method tests conducted in several studies could not find any relation between surface characteristics and modes of fracture [31][32]. Both types of fracture caused dimple configurations. Fatigue was interpreted as portions failed in ductile manner when the cross-section was no longer able to tolerate the load (Figure 2A, Figure 2B). In ductile fractures, dimples followed different patterns of equiaxial or skewed appearance depending on the state of the stress. Voids were a common view in all the cases of torsional failures. Elongation of dimples between voids (tearing ridges) was the specific characteristic in shear cases. Plastic deformation was seen as either necked region across the fracture surface or macroscopically deformed margins adjacent to the fracture surface.

**Table 1:** Mean (±SD) rotations to failure (NCF) for Race and ProTaper files at all tested geometries.

****	**Radius, Angle**	**5mm, 60°**	**5mm, 30°**	**2mm, 60°**	**2mm,30°**
**ProTaper (F1)**	Torsional Fraction	10	3	10	10
	Fatigue Fraction	-	7	−	−
	NCF (Mean±SD)	184.5±29.8	403.2±46.2	79.8±19.2	202.5±47.2
**RaCe (25/.06)**	Torsional Fraction	10	−	10	10
	Fatigue Fraction	−	10	−	−
	NCF (Mean±SD)	184.5±29.8	403.2±46.2	79.8±19.2	202.5±47.2

A school of thought claims that electropolishing makes the surface of NiTi instruments more resistant to fracture [27][33] (Figure 2C, Figure 2D). However, our study demonstrated that electropolishing cannot affect the core where most of the mechanical properties reside (at 5mm radius and 60⁰ curvature), concurring with another study [33].

No significant differences could be detected between RaCe and ProTaper at 2mm radius and 60° canals where failures occurred in torsional mode. SEM observations indicated 26 sheer failures in the ProTaper group, but only 10 in the RaCe group. However, the core of an instrument seems to be a better determinant factor at more acute-angled curvatures, where more severe loads can be generated to skew rotary instruments [34].

Files had significantly lower fracture resistance in canals with more acute angles and low radius of curvature. An increase in canal curvature led to greater compression and tensile forces on the file and subsequently less NCF, which is consistent with Pruett et al.’s findings [14].

The ratio for fatigue-to-torsion incidence in clinically separated instruments was reported to be 2:1 [10][11]; whereas some studies have reported that torsional failure is the predominant mode [4][35]. Torsional failure was more important in the present study. Low-cycle fatigue process needs at least 10(4) cycles of stress to occur [27]. Thus, less rotating cycles in rotary instruments with low-speed handpieces is insufficient to create obvious fatigue features. In the present study mild signs of fatigue (Figure 2A, Figure 2B) were detectable in the instruments having the highest NCF values. Canal geometry/morpgology can affect the magnitude of stress on instruments [9]. It seems that huge stresses generated by severe curvatures created the greatest ductile failures.

Ductile fracture is usually preceded by a localized decrease in the diameter of the instrument; this is called necking. Necking begins after the ultimate stress (the point that stress reaches the maximum level). So a neck is formed as the instrument is further elongated (Figure 2E). The formation of a neck introduces a triaxial state of stress in the region. A hydrostatic component of tension acts along the axis of the specimen at the center of the necked region. Many fine inclusions form in this region and grow and coalesce into a central crack. This crack grows in a direction perpendicular to the axis of the instrument until it approaches the surface of the instrument. The central crack which forms early tends to concentrate the deformation at its tip in narrow bands of high shear strain. Sheets of voids are nucleated in these bands and the voids grow and coalesce into the local fracture of the sheet (Figure 2F) [27]. Generally, the plane of the fracture is in normal appearance to the longitudinal axis (Figure 2I). In addition, large numbers of fairly small pieces are detected on the fractured surface (Figure 2K). In the current study, fractures started on a plane of maximum shear stress parallel to the axis of the instrument [27] (Figure 2L).

The more prevalent sites for void formation are inclusions or fine oxide particles. The higher rate of tensile fracture and its related void sheet formation in ProTaper files may relate to some metallographic aspects [27].

Since the shear stress values increase peripherally [27], any increase in the cross-sectional area increases the volume of the instrument. This will enlarge the core volume and result in more stiffness of the instrument. In addition, 3 shear failures of ProTaper files, which were recorded in gentle curvature of 5mm and 30° show that the etiology cannot be related to the sharp curvatures only. It seems that a structural defect such as numerous microvoids has guided the load components along the instruments’ axis. The longitudinal cracks which occurred commonly on the ProTaper instruments with shear failure (Figure 2L) may indicate that ProTaper instruments are more vulnerable to torsional stress compared to RaCe instruments.

Although lubricant (PBS) was used continuously during instrument rotation, it seems that the friction coefficient did not decrease. The high friction coefficient between stainless steel and NiTi instruments resulted in greater engagement of the instrument with the simulated canal walls and in more torsional failures.

## CONCLUSION

Both an increase in the angle and a decrease in the radius of curvature resulted in decreased cyclic fatigue resistance of the two file systems. Torsional failure was the predominant mode of fracture for the RaCe and ProTaper systems. However, this is an in vitro study and the conditions do not emulate the properties of dentine or enamel.
